# LEGO^®^ Bricks as Building Blocks for Centimeter-Scale Biological Environments: The Case of Plants

**DOI:** 10.1371/journal.pone.0100867

**Published:** 2014-06-25

**Authors:** Kara R. Lind, Tom Sizmur, Saida Benomar, Anthony Miller, Ludovico Cademartiri

**Affiliations:** 1 Department of Materials Science & Engineering, Iowa State University, Ames, Iowa, United States of America; 2 Ames Laboratory, US Department of Energy, Iowa State University, Ames, Iowa, United States of America; 3 Department of Agronomy, Iowa State University, Ames, Iowa, United States of America; 4 Department of Chemical & Biological Engineering, Iowa State University, Ames, Iowa, United States of America; Freie Universität Berlin, Germany

## Abstract

LEGO bricks are commercially available interlocking pieces of plastic that are conventionally used as toys. We describe their use to build engineered environments for cm-scale biological systems, in particular plant roots. Specifically, we take advantage of the unique modularity of these building blocks to create inexpensive, transparent, reconfigurable, and highly scalable environments for plant growth in which structural obstacles and chemical gradients can be precisely engineered to mimic soil.

## Introduction

Microfluidics[1], and other engineered environments[5], [6] can produce highly controlled micrometer-scale environments for the study of organismal model systems (e.g., mammalian cells). However, scientists or engineers interested in manipulating the environment of cm-scale organisms (e.g., plants) have remarkably few convenient tools at their disposal[7], [8]. This paucity is partly due to the demanding design requirements associated with larger scales (e.g., cost). This liability is particularly evident in the study of plants and their root systems.

The development of plants in soil is an important subject of investigation. The provision of food to the global human population is under severe pressure (our supply of food is predicted to be far below demand by 2050[9]) and depends on plant roots[10] (97.6% of global calorie consumption is derived from plants[11]). Roots influence a plant's yield and whether a plant will survive stresses. We know that root growth is strongly affected by its environment, soil, but our mechanistic understanding of these effects is imperfect[10], [12] and strongly limited by technical challenges.

Root development is a difficult process to study experimentally. (i) Plants display highly variable root systems, even when genetically identical[13]. (ii) Roots are remarkably sensitive to a variety of stimuli (e.g., gravity, light, touch, moisture, nutrients, oxygen, temperature, trauma, electric fields[14]). (iii) Any volume of soil is unique and impossible to replicate exactly[15], [16]. (iv) Its heterogeneity makes it opaque to most forms of radiation[17]. (v) Its structural and chemical characteristics (i.e., porosity, surface chemistry, nutrient gradients, oxygen gradients, bulk composition, soil biota) cannot be independently manipulated.

One approach to avoid this complexity is to characterize the growth of plants in soil-less media, e.g., hydrogels, paper, glass beads, sand. These systems are less inhomogeneous and irreproducible than soil and can be modified – usually to a limited extent – to mimic soil properties such as chemical composition [18], physical structure [19], [20], water availability [21], refractive index [22], or mechanical strength [23]. However, the lack of modularity, versatility, structural precision, and the very limited control over structural and chemical heterogeneities in these systems severely limits the type, complexity, and reproducibility of the experiments they can perform. Microfluidic approaches offer fascinating capabilities for the study of plant roots, but are subjected to limitations in their throughput and in the size of the plants they can host [4], [24], [25].

We here demonstrate that LEGO bricks are highly convenient and versatile building blocks for building cm-scale engineered environments for plant roots. Their modularity enables the fabrication of environments with highly controlled structural and chemical heterogeneities that are suitable for convenient quantitative studies of environmental effects on plant phenotypes[26].

## System Design

A convenient experimental platform for the study of root development in controlled environments must satisfy a demanding set of design constraints. LEGO bricks, while conceived and sold as toys, satisfy these constraints.

### Modularity

Modular systems can produce many structurally distinct environments from a few different components. Features can be added or removed without remanufacturing the entire experimental setup. LEGO structures are modular. The smallest bricks are 8*x*8*x*6 mm. The largest are 48*x*8*x*50 mm. The number of different structures that can be made with these units is staggering: six identical bricks can form almost a billion different structures[27].

### Scalability

Confinement can affect the physiology of an organism[28]. The ability to create experimental platforms of a range of sizes enables researchers to study any plant and their ensembles. LEGO structures can be easily scaled to accommodate different plant species: the smallest enclosed environment that can be produced with LEGO bricks measures 0.35 cm^3^ in volume, and it is theoretically possible to create LEGO structures capable of containing the largest plant species.

### Structurally precise

Roots are sensitive to the physical structure of their environment. For example, the study of root thigmotropism (the response of a root to touch) requires structures that are of an exact size and shape. The molds used to produce LEGO bricks are accurate to within 5 µm[29], which is comparable to the diameter of a root hair and to the resolution of 3D printing (minimum layer thickness is ∼50 µm in some of the best current models).

### Capable of increasing levels of complexity

A good model system allows for the controlled introduction of experimental variables. LEGO bricks can be used –as shown below – for the generation of physical barriers, air pockets, chemical gradients, and interconnecting chambers to control the growth environment of a plant.

### Simplicity

Simple setups reduce the risk of operator-induced systematic errors. Differently from microfluidic approaches, the assembly of structures from LEGO bricks does not require technical training so undergraduate students can perform LEGO brick-based plant experiments from their first day in the laboratory. Simple experiments that demonstrate fundamental principles of plant growth (e.g., tropisms) or encourage experimental creativity can be conducted by school children of all ages during science education classes[30].

### Reproducibility

Plant root experimental platforms (e.g. sand columns, rhizotrons, split-root pots) are typically made from scratch. Their reproducibility between labs or across continents cannot be guaranteed. The unique selling point of LEGO bricks is that bricks bought in separate batches are essentially identical and backward- and forward-compatible with each other. Experiments created from LEGO bricks can be accurately replicated anywhere in the world.

### Affordability

The more expensive each experiment is, the fewer experiments can be conducted with finite resources. This fact is especially meaningful in developing nations[31] and in research fields, like plant science, where throughput is an essential parameter. Individual LEGO bricks cost between $0.10 and $1.00 and are sold worldwide. A LEGO structure capable of growing a plant costs $3.1 and is reusable: some LEGO bricks in our lab have been in near-constant use for two years.

### High throughput

**Figure 1:**
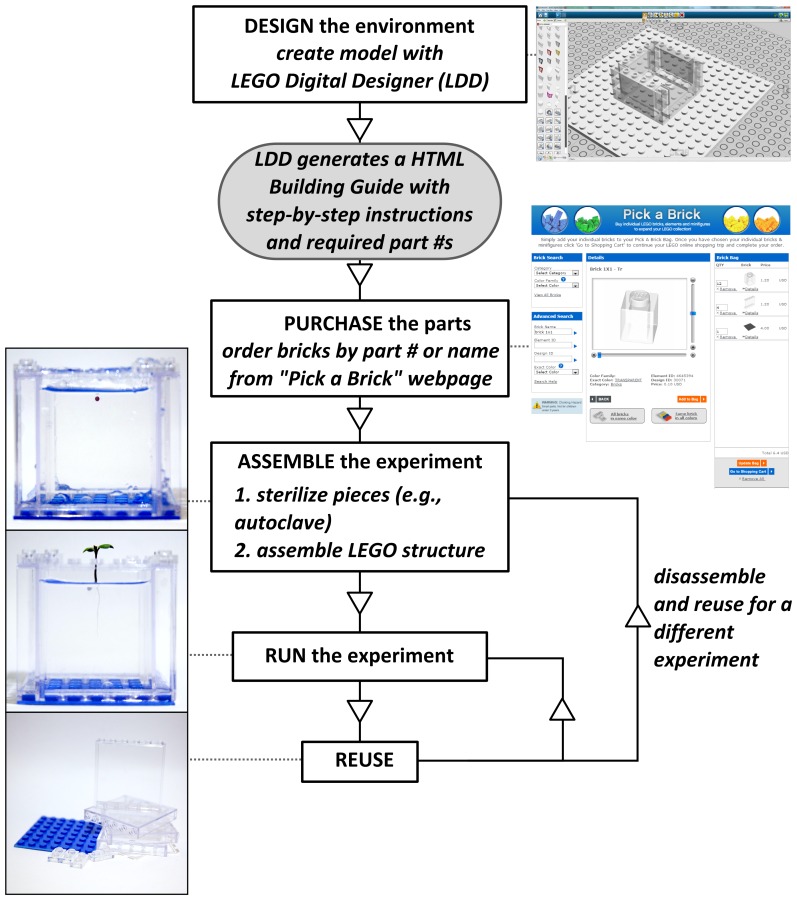
Scheme of the process of carrying out a plant growth experiment using LEGO bricks as building blocks. The same process can be used to prototype and fabricate other biological experiments.

The ability to run a large number of experiments at the same time is essential for the establishment, for example, of genotype-environment-phenotype relationships[32]. A LEGO structure like the one shown in Figure 1 can be assembled in less than a minute.

### Transparency

Twenty eight different LEGO bricks are made from transparent polycarbonate which can be assembled into transparent structures for the real-time monitoring of plant roots over time.

### Autoclavable

Tissue cultures require sterile conditions. Transparent LEGO bricks (with the exception of large base plates) are autoclavable due to their polycarbonate composition: they still fit together in the same way as they do prior to autoclaving and are still transparent after more than 50 autoclave cycles. Opaque LEGO bricks are made from acrylonitrile-butadiene-styrene block copolymer (ABS), and can be sterilized with ethanol or bleach.

### Three-dimensionality

While 2D platforms offer significant advantages in terms of visualization and practicality[33], 3D mediums are more representative of the natural environment of roots[34]. LEGO bricks allow for the creation of nearly arbitrary 3D structures.

### Chemical inertness

Legislative standards ensure the safety to children of LEGO bricks sold in the USA and EU. These standards include maximum soluble levels of toxic or hazardous substances.

### Compatibility with existing growth environments

Tools that integrate with existing experimental platforms are often the most useful. The modularity of LEGO structures enables them to integrate with laboratory protocols e.g., LEGO structures can hold gel, beads, sand, soil, 3D-printed elements, or be structurally precise elements in other setups[35].

## Results and Discussion

Figure 1 shows a flow diagram of the design, assembly, disassembly, and re-assembly of an experiment based on LEGO bricks. The website of the LEGO Group (www.lego.com) provides a free software (LEGO Digital Designer, LDD) for the CAD-like design of structures using any available LEGO brick. The software outputs a step-by-step assembly guide and a list of the required parts. Individual bricks can be purchased through the “Pick a Brick” section of www.lego.com or other outlets (e.g. local LEGO stores, EBay). Sterilization of LEGO bricks can be performed before or after assembly. The preservation of sterility requires the structure to be maintained in a sterile container during the course of an experiment.

The simplest example of a plant germination and growth environment based on LEGO bricks is shown in Figure 1. The LEGO bricks are assembled into a container that contains a root growth medium on which a seed is germinated and grown: Figure 1, for example, shows a *Brassica rapa*, Wisconsin Fast Plant, Astroplant, *dwf1*, growing on a transparent hydrogel, Gellan gum. While gel media for root growth are very commonly used in experiments[26], they are not the best mimic of soil: root architectures grown in an homogeneous media will not match those of plants grown in real soil[36]. However, gel media allows us to demonstrate three essential capabilities of LEGO-based biological environments: their ability to hold liquids, their compatibility with real-time observation and root structure analysis, and their use in generating reconfigurable environments that include controlled heterogeneities. Furthermore, LEGO environments are not limited to gel media: the environment shown in Figure 1 can hold other media of choice, e.g., sand, perlite, soil.

**Figure 2:**
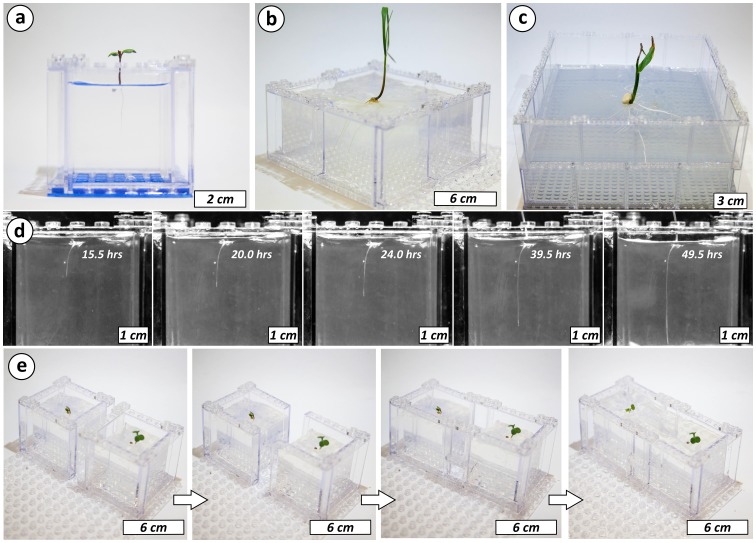
Versatility, transparency, and modularity of the LEGO-based environments for plant growth. a-c) pictures of basic LEGO-based environments growing Fast Plants, Wheat and Corn. The size of the environments can be controlled to match the size of the organism under consideration. d) Timelapse imaging of *Lepidium sativum* root development through the walls of a LEGO-based environment. The images indicate the time since germination. e) Examples of a LEGO-based system that allows for the dynamic change of the environment of a plant. Two plants (Fast Plants) are grown in isolated environments. The environment is then modified, during growth, to allow the two plants to share the same environment and interact.

Since structures built from LEGO bricks are not waterproof, their use to hold gels requires some stratagems (see Supporting Information for details and Movie S1 for a demonstration). The LEGO structure must be chilled in a freezer before the cool gel solution is poured in it just prior to setting. Using this approach, leakage of the gel solution was minimal. These basic environments can be easily scaled to match the dimensions of the organism under consideration and the time the organism is allowed to grow. Figures 2a, 2b, and 2c show the use of LEGO bricks to create containers with very different dimensions (5×5×5 cm, 10×10×5 cm, and 20×20×10 cm) for the growth of Fast Plants, *Triticum polonicum* (Wheat), and *Zea mays* (Corn).

The transparency and flat walls of LEGO bricks allows for good quality real time imaging of the development of the root system. Figure 2d shows time-lapse imaging of *Lepidium sativum* (Garden cress) roots over the course of ∼48 hrs from germination in a LEGO-based environment. The plant was chosen for its relatively fine roots (∼350 µm thickness) that would have been hard to image in a poorly transparent system.

**Figure 3:**
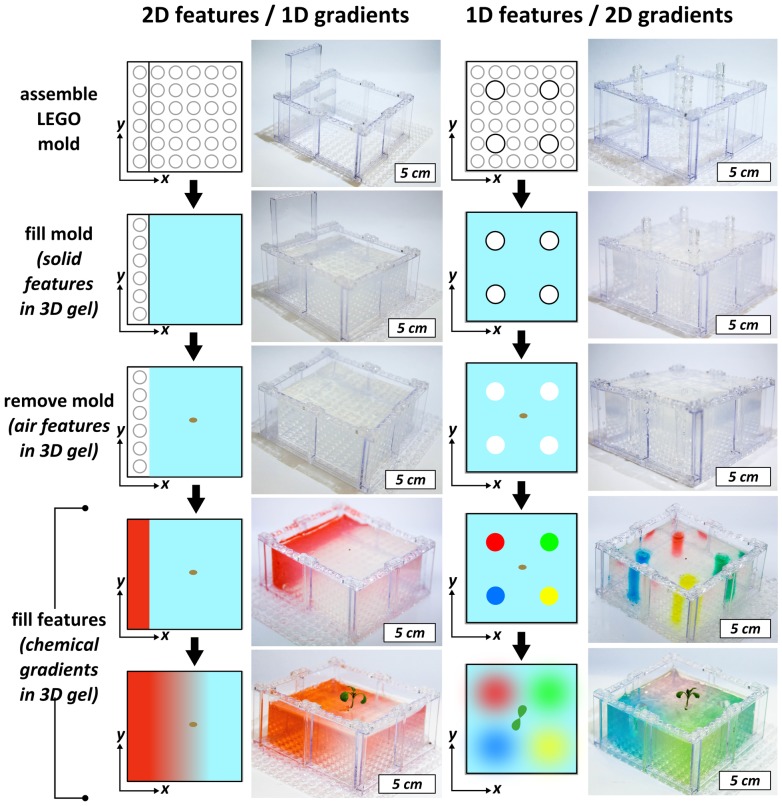
Fabrication of controlled heterogeneities in plant growth environments. Sequence of diagrams and corresponding images illustrating the generation of a 1D and 2D heterogeneities (solid features, air pockets, and chemical gradients) across a developing root system of a Fast Plant. In the bottom panels, the red linear gradient is of MS nutrients (dye is added for visibility), while the radial gradients are from potassium phosphate (green), potassium nitrate (yellow), calcium chloride (red), and magnesium sulfate (blue).

The reversible nature of the mechanical bond between the bricks provides two important capabilities: the creation of reconfigurable biological environments, and of highly controlled heterogeneities (i.e., solid obstacles, air pockets, and chemical and soil biota gradients) in an otherwise homogeneous growth medium. Figure 2e demonstrates a reconfigurable plant growth environment. Two Fast Plants were grown in gel in separate containers assembled on the same base plate. The LEGO brick walls separating the two containers were removed and reconfigured to make one larger container. The volume separating the two plants was then filled with more gel, fluidically connecting the two plants. Figure 3 demonstrates the generation of controlled heterogeneities in a homogeneous gel medium for plant growth by a simple templating strategy borrowed from the materials science “toolbox”. A gelling mixture was poured into a LEGO-based mold. LEGO-based features in the mold can be used as solid heterogeneities to study the physical interaction of plant roots with solid objects (thigmotropism). After gelation, LEGO-based molds could be removed, leaving behind precisely positioned air pockets that would serve as sources of oxygen gradients into the gel. These pockets could be then refilled with a hydrogel containing a desired chemical to generate precisely positioned one-dimensional (Figure 3, bottom left panel) or two-dimensional (Figure 3, bottom right panel) nutrient gradients. The above process can be combined to create environments with solid heterogeneities, air pockets (i.e., oxygen gradients), and chemical (e.g., nutrients, toxins, signaling molecules) gradients simultaneously (see Appendices S1).

## Conclusions

In summary we demonstrated that LEGO-based environments can (i) scale to the size of the organism under consideration, (ii) allow for real time monitoring of root systems in 3D, (iii) be structurally reconfigured to change the environment of an organism during its development, and (iv) generate precisely controlled heterogeneities (i.e., solid barriers, air pockets, chemical and soil biota gradients) in an otherwise homogeneous growing medium.

This manuscript also proposes a broader concept: the use of reusable and mechanically interlocking building blocks for the construction of biological environments for cm-scale organisms and systems of organisms. Modular and reusable building blocks can alleviate the challenges associated with the large scales of plant science experiments, while providing new capabilities (e.g., controlled heterogeneities, reconfigurable environments) for the study of environmental effects on biosystem development. Furthermore, this concept provides materials chemists and engineers with two stimulating opportunities: (i) to creatively engage with the synthesis or development of increasingly capable cm-scale biological environments for important organisms such as plants, and (ii) to use these environments to test hypothesis concerning plants that are compatible with their skillset. Compelling opportunities lie in extending our approach to chemically synthesized bricks, LEGO-compatible 3D-printed bricks and objects, and commercial bricks from other manufacturers. Our laboratory will be introducing a set of integrated tools for the fabrication of frugal but sophisticated[37] cm-scale environments for the study of plants and other organisms[35].

## Supporting Information

Appendices S1 Materials, methods, and procedures for the generation of (i) basic LEGO-based environments, (ii) LEGO-based environments with linear chemical gradients, (iii) LEGO-based environments with cylindrical chemical gradients, (iv) larger scale LEGO-based environments. Demonstration of a LEGO-based environment combining controlled obstacles, air pockets, and multiple chemical gradients. Calculation of the smallest possible LEGO-based environment. Limitations, open questions, and failed experiments.
(PDF)

Figure S1 Summary snapshots of the assembly of a basic LEGO-based plant growth environment.
(TIF)

Figure S2 Summary snapshots of steps for root analysis using WinRhizo of two *brassica rapa* roots grown in LEGO-based plant growth environment.
(TIF)

Figure S3 Snapshots of the procedure to produce linear features (solid obstacles, air pockets and chemical gradients) in a homogeneous gel by using LEGO bricks.
(TIF)

Figure S4 Snapshots of the procedure to produce 2-dimensional features (solid obstacles, air pockets and cylindrical chemical gradients) in a homogeneous gels by using LEGO bricks.
(TIF)

Figure S5 Photograph of a 3D plant growth environment based on LEGO bricks featuring three different types of heterogeneities: a solid barrier (top left), an air pocket (bottom right) and two different cylindrical chemical gradients (top right and bottom left).
(TIF)

Figure S6 Depiction of the smallest LEGO-based environment.
(TIF)

Movie S1 Assembly of a basic LEGO-based environment.
(M4V)

Movie S2 Plant harvesting procedure from a basic LEGO-based environment.
(M4V)

Table S1 Preparation of salt solutions for cylindrical dye experiment.
(PDF)
