# Supplementary material for: LEGO® Bricks as Building Blocks for Centimeter-Scale Biological Environments: The Case of Plants
Source: PLoS One. 2014 Jun 25;9(6):e100867. doi: 10.1371/journal.pone.0100867 (PMC4071033; doi:10.1371/journal.pone.0100867)
Supplement: Appendices S1 — Materials, methods, and procedures for the generation of (i) basic LEGO-based environments, (ii) LEGO-based environments with linear chemical gradients, (iii) LEGO-based environments with cylindrical chemical gradients, (iv) larger scale LEGO-based environments. Demonstration of a LEGO-based environment combining controlled obstacles, air pockets, and multiple chemical gradients. Calculation of the smallest possible LEGO-based environment. Limitations, open questions, and failed experiments. (PDF) [file pone.0100867.s001.pdf]

LEGO® Bricks As Building Blocks For Centimeter-Scale Biological Environments: The Case Of Plants

KARA R. LIND†, TOM SIZMUR†‡§, SAIDA BENOMAR†‡, ANTHONY MILLER†‖, LUDOVICO CADEMARTIRI†‡⊥*

† Department of Materials Science & Engineering, Iowa State University, Ames, IA, 50011
‡ Ames Laboratory, US Department of Energy, Iowa State University, Ames, IA, 50011
‖ Department of Agronomy, Iowa State University, Ames, IA, 50011
⊥ Department of Chemical & Biological Engineering, Iowa State University, Ames, IA, 50011
§ current address: Department of Sustainable Soils and Grassland Systems, Rothamsted Research, Harpenden, Heartfordshire, AL5 2JQ, United Kingdom

Author to whom correspondence should be addressed: lcademar@iastate.edu

APPENDICES

MATERIALS

Reagents

- Murashige & Skoog basal salt mixture with vitamins (PhytoTechnology Laboratories cat. no. M519)
- Phytagel (Sigma Aldrich cat. no. P8169)
- Agar (PhytoTechnology Laboratories cat. no. A111)
- Sodium Chloride crystalline (Fisher Scientific cat. no. S671-3)
- Calcium Chloride dehydrate (Fisher Scientific cat. no. C79-500)
- Potassium Phosphate monobasic (Sigma Aldrich cat. no. P5655)
- Potassium Nitrate (Sigma Aldrich cat. no. P8291)
- Magnesium Sulfate anhydrous (Sigma Aldrich cat. no. M7506)
- Bleach (Chlorox concentrated bleach 8.25% sodium hypochlorite)
- Milli-Q deionized water (produced by a Milli Q synthesis water system with a resistivity of ≥18.2MΩ·cm)
- Ethyl alcohol (200 proof no. 64-17-15)

Equipment

- Class II Biosafety Cabinet (Nuaire model 425-440)
- Autoclave (Primus Sterilizer model PSS5)

- 1 Liter glass bottles with cap (Sigma Aldrich cat. no. CLS13951L)
- 20ml glass vials with lids
- Analytical balance
- sterile pipette 50-200 µl, sterile pipette tips
- Microwave
- Digital Camera (Canon 50D, 100 Macro lens)
- Freezer (4 °C)

Apparatus and Consumables

Magenta vessel GA-7 77 mm × 77 mm × 97 mm (Sigma Aldrich cat. no. V8505)
Sterilite flip top vessel 19.4cm L x 16.5cm W x 11.4cm H (Sterilite cat. no. 1803)
Storage Bin 15-5/8"L x 13-1/8"W x 6-3/4" H (Container store cat. no. 10008761)

Wall Element – TR 1X2X3 (LEGO Element ID: 6010737)
Wall Element – TR 1X2X2 (LEGO Element ID: 4113028)
Wall Element – TR 1X4X3 (LEGO Element ID: 4558208)
Wall Element – TR 1X6X5 (LEGO Element ID: 4504229)
Plate 1X2 (LEGO Element ID: 4167842)
Plate 8X8 (LEGO Element ID: 4210802)
X-large plate (LEGO Element ID: 4294466)
Round brick (LEGO Element ID: 3006840)

Seeds: *Brassica rapa* seeds (Wisconsin Fast Plants; Astroplants, *dwf1*), *Lepidium sativum*, *Brassica oleracea* (Broccoli), *Solanum lycopersicum* (tomato), *Arabidopsis thaliana, Nicotina tabacum* (Tobacco), *Zea mays* (Corn) and *Triticum polonicum* (Wheat).
Tweezers
Petri dish
Aluminum foil
Autoclave indicator tape
Nitrile gloves
Food coloring (red, green, blue, yellow colors)
Parafilm
Whatman 1 chromatography paper
Growth chamber (we manufactured custom-made growth chambers out of wood and aluminum foil. The purpose of our growth chambers was to ensure a homogeneous illumination of the plants and avoid air drafts. The growth chambers are in the form of a parallelepiped with a wooden framework and aluminum foil walls. An LED panel hangs from the ceiling and provides 9000 lumens of visible light to the growing plants.)

METHODS

Reagent setup

1. **Gel Nutrient solution**
   Dissolve 2.215 g of Murashige & Skoog and 3 g of Phytagel in 1 Liter of Milli-Q water. Sterilize this solution by autoclaving (sterilization temperature 121 °C)

2. **70 % ethanol (vol/vol)**

Add Milli-Q water to 70 ml of ethanol and bring solution to final volume of 100 ml.

3. **Agar nutrient for seeds**
   Dissolve 50mg of Agar and 22.15 mg Murashige & Skoog in 10 ml (0.5%) of Milli-Q water in a 50 ml glass vials (50mg/ml = 0.5%). Sterilize by autoclaving (sterilization temperature 121 °C).

4. **Saturated NaCl solution**
   Dissolve 500 grams of NaCl in 1 Liter of Milli-Q water. Sterilize by autoclaving (sterilization temperature = 121 °C).

5. **Seed sterilization**
   For *Zea mays, Triticum polonicum, Arabidopsis thaliana*, and *Nicotina tabacum* experiments, prepare 45 ml of dilute bleach. Combine 5 ml of bleach with 40 ml of Milli Q water in a centrifuge tube. Place seeds in Petri dish and pour 15 ml of dilute bleach solution on top of seeds. Incubate for 15 minutes before washing seeds with sterile water. For *Zea mays* and *Triticum polonicum*, place seeds between two sheets of Whatman 1 filter paper cut to size of a new sterile Petri dish. Secure Petri dish with Parafilm. Store *Zea mays* and *Triticum polonicum* at 4 °C for 48 hours before use.
   For *Arabidopsis thaliana* and *Nicotina tabacum*, place seeds in Gibberelic acid (1mg GA /ml sterile di water) on sterile Whatman 1 paper cut to fit Petri dish. Secure Petri dish with parafilm and store at 4°C for at least 24 hours before use.
   For *Brassica rapa, Lepidium sativum, Brassica oleracea, Solanum lycopersicum, and Rosmarinus officinalis* experiments, add 15 ml of 70% ethyl alcohol to cover seeds in Petri dish. Incubate for 15 minutes then wash with sterile DI water or sterile nutrient solution without phytagel. This solution is prepared by adding 2.215 g of Murashige & Skoog basal salt with vitamins in 1 liter of MilliQ water. Solution must be autoclaved before used for seed washing.

PROCEDURES

<u>Steps 1-8: Basic LEGO-based environment for plant growth</u>

<u>Steps 1-3: Assembly of plant vessel</u>

1. For each Magenta vessel plant experiment, gather 6 LEGO 1X2 plates, 4 LEGO 1X6X5 wall elements, and 1 LEGO 8X8 plate. Carefully cut with scissors the 8X8 plate so that it is 7X7. Note: LEGO references LEGO piece sizes by the raised dots on each piece and this is how we reference them.
2. Attach wall elements concave side outward in a box shape using one 1X2 plate to connect walls together. Place connected walls in Magenta vessel and add a small piece of autoclave indicating tape to lid and autoclave at 121°C to sterilize. For 7X7 plate sterilize by soaking in dilute bleach solution same concentration as that used for seed sterilization. Rinse with DI water and spray with 70% ethanol and introduce into biosafety cabinet.
3. Introduced autoclaved Magenta vessels into biosafety cabinet by spraying with 70% ethanol. Once in biosafety cabinet, remove attached wall structure. Attach 7X7 LEGO plate to walls creating a LEGO box. Place back in Magenta vessel and store at 4 °C for at least 2 hours or just prior to pouring nutrient gel.

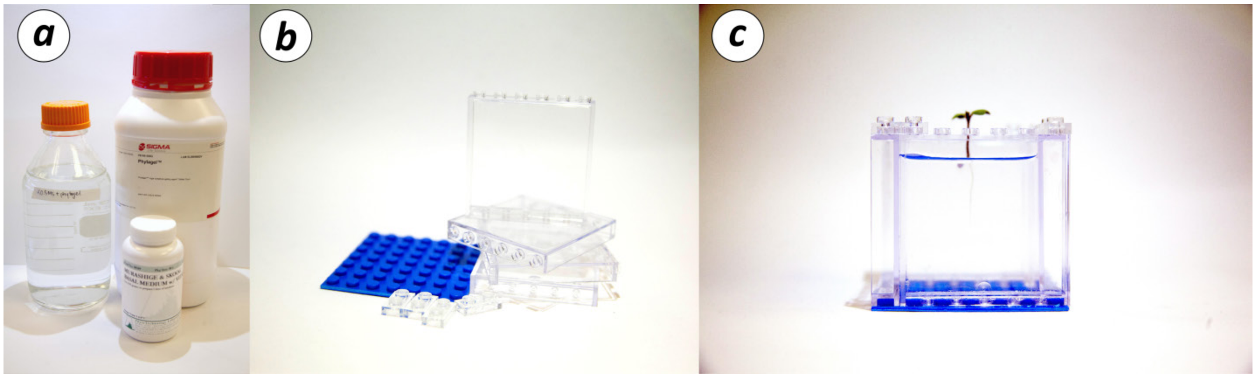

Figure S1. Summary snapshots of the assembly of a basic LEGO-based plant growth environment.

<u>Steps 4-5: Introducing nutrient gel into plant vessel</u>

4. Once nutrient gel has cooled to 40 °C after autoclaving, spray bottles with 70% ethanol and introduce into biosafety cabinet. We have found it takes about 150 minutes for gel to cool to 40°C. Remove Magenta vessels from 4°C storage once gel has cooled to 35°C and spray with 70% ethanol before placing in biosafety cabinet.

5. Remove the LEGO box from Magenta vessel and slowly pour gel into bottom. Once the gel has sit for a few minutes begin to pour more into the box to about the halfway point. Wait a few more minutes before continuing. It is almost certain that the cracks will allow for some of the gel to escape the box. Filling the box with gel is the most complicated part of the experiment and requires patience. As the gels cools to 31 °C the gel will begin to set (gelling temperature 27-31 °C). To ensure a firm gel, the boxes are placed back inside the Magenta vessels and then kept in the biosafety cabinet to allow to set for a minimum of 3 hours. In most cases, we wait overnight.

6. An optional step would be to include a super-saturated sodium chloride solution at the bottom of the LEGO box for humidity control. If required 2 1X4X3 LEGOs should be included in the autoclave cycle inside the Magenta box. For the saturated salt solution, 500 g of sodium chloride is dissolved in 1 liter of Milli-Q water at room temperature. Sodium chloride should be added to the point where it no longer dissolves.

<u>Steps 7-8: Planting the seed</u>

7. For *Zea mays* and *Triticum polonicum*, the seed is placed with a sterile tweezers on top of the set gel and then covered with 500 μl of agar if *Zea mays* or 200 μl of agar if *Triticum polonicum* using a pipette. For all other seeds, the seed can be placed into the nutrient gel using a sterile tweezers.

8. The Magenta vessel lid is reattached and removed from the biosafety cabinet. The assembled experiment can then be placed in the growth chamber.

<u>Steps 9-14: Harvesting plant (see Movie S2)</u>

9. When the experiment is complete, the harvesting procedure can begin. First the Lego box is imaged on each side focusing on the root structure using a digital camera.

10. If desired, the Lego walls and Lego baseplate can be removed as the gel will remain intact. The roots can then be imaged an additional time from the desired angles.

11. Gently toss the gel cube containing the plant against the bottom of a vessel. Root growth produce fractures in the gel. Mechanical stress on the gel, as that caused by tossing, causes the propagation of those fractures in a way that preserves the integrity of the root system. Remove the plant from the broken gel and rinse the roots with DI water to remove any remaining gel.

12. Absorb as much water from the roots using a paper towel. Then place the plant on a paper of known size. We find that black paper works best for root analysis.
13. Separate the roots from each other using tweezers. Image the whole plant and paper support.
14. Remove the shoot with scissors and image the roots again.

Steps 15-16: Using WinRhizo to obtain structural root data

15. Take the image of the whole root system on a paper support of known size and convert it into a binary (i.e., black and white) image.
16. Using WinRhizo, determine morphological information (e.g., root length, root volume).

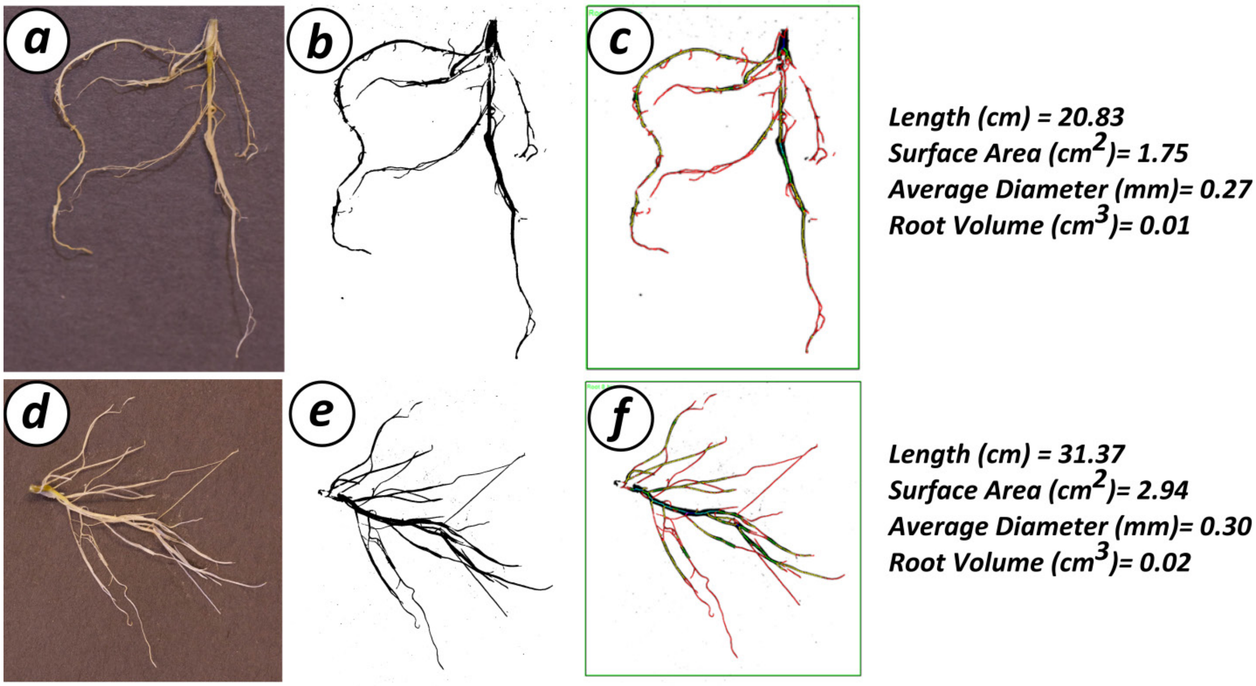

Figure S2. Summary snapshots of steps for root analysis using WinRhizo of two *brassica rapa* roots grown in LEGO-based plant growth environment.

Steps 1-5: LEGO-based environment with linear chemical gradient

1. A LEGO box is constructed using 8 1X6X5 wall element LEGOs, 8 1X2 plates, and 1 16X16 LEGO baseplate (we advise using a translucent or light gray color). Additionally 2 1X6X5 wall elements and 2 1X4X3 wall elements are used for the gap for the linear gradient as shown below.
2. The attached walls are autoclaved in plastic container in which the experiment will be done. The baseplate is sterilized using dilute bleach and then followed by spraying of 70% ethanol before placing in the biosafety cabinet. Once everything is sterilized the baseplate is attached to the walls in the autoclaved container and put in the freezer for at least 2 hours.

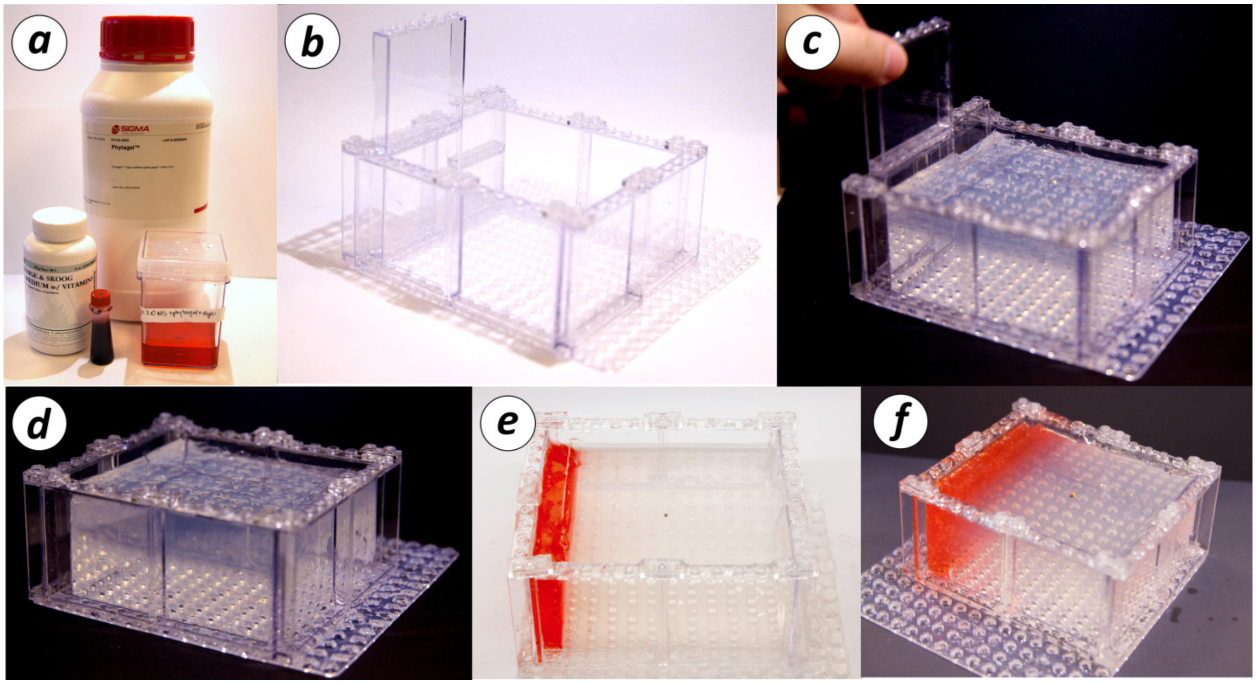

Figure S3. Snapshots of the procedure to produce linear features (solid obstacles, air pockets and chemical gradients) in a homogeneous gel by using LEGO bricks.

3. The gel nutrient matrix prepared the same as before using 0.5 MS and 3 g/ Liter phytagel. Additionally for the dye linear experiment, a solution 100 ml solution is prepared with 443 mg of MS, 300 mg phytagel, 200 µl red food coloring in 100 ml of milliQ water.
4. The dyed gel solutions and nutrient matrix can be autoclaved using the liquid cycle. Typically, the dyed solution is autoclaved the following day in order to let the nutrient gel set before removing the walls used for the linear gradient.
5. At this point, the LEGO box is cold from being the freezer for at least 2 hours. Spray the plastic box with 70% ethanol and reintroduce into the biosafety cabinet. Once the gel reaches near 35 °C slowly pour the gel in the LEGO box. It works best to add a small amount of gel at a time. The gel required for this experiment is around 600 ml. Allow the experiment to remain in the biosafety cabinet overnight to allow the gel to fully set. The next day, the walls for the gradient are carefully removed. If the walls are stuck to the baseplate, a tweezers can be used to aid in the removal. At this point the dyed solution that was prepared can be added to the opening or if doing a gas study they can remain empty. For the dye study, 30 ml of dyed solution is added to the hole. For either the dye experiment or gas experiment, the seed (Brassica Rapa in our case) can be planted into the center of the gel. Parafilm is used to seal the container before placing in the growth chamber.

Steps 1-8: LEGO-based environment with cylindrical chemical gradient

1. A LEGO box is constructed using 8 1X6X5 wall element LEGOs, 8 1X2 plates, and 1 16X16 LEGO baseplate (we advise using a translucent or light gray color). Additionally LEGO columns are built using 7 round brick LEGOs for each column.
2. The attached walls and columns are autoclaved in plastic container in which the experiment will be done. The baseplate is sterilized using dilute bleach and then followed by spraying of 70% ethanol before placing in the biosafety cabinet. Once everything is sterilized the baseplate is attached to the walls in the autoclaved container and put in the freezer for at least 2 hours.

3. The gel nutrient matrix is prepared as detailed in the previous procedure using 0.5 MS and 3 g/l phytagel. Additionally for the dye cylindrical experiments, 10mg/ml salt solutions are prepared with additional dye according to the following table:

Solution #	Salt	Additional components	Dye
**1**	100 mg potassium phosphate	10 ml milliQ water<br>30 mg phytagel	Green food coloring<br>30 µl
**2**	100 mg potassium nitrate	10 ml milliQ water<br>30 mg phytagel	Yellow food coloring<br>30 µl
**3**	100 mg calcium chloride	10 ml milliQ water<br>30 mg phytagel	Red food coloring<br>30 µl
**4**	100 mg magnesium sulfate	10 ml milliQ water<br>30 mg phytagel	Blue food coloring<br>20 µl

Table S1: Preparation of salt solutions for cylindrical dye experiment.

The dyed solutions and nutrient matrix can be autoclaved using the liquid cycle. Typically, the dyed solutions are autoclaved the following day in order to let the nutrient gel set before removing the columns.

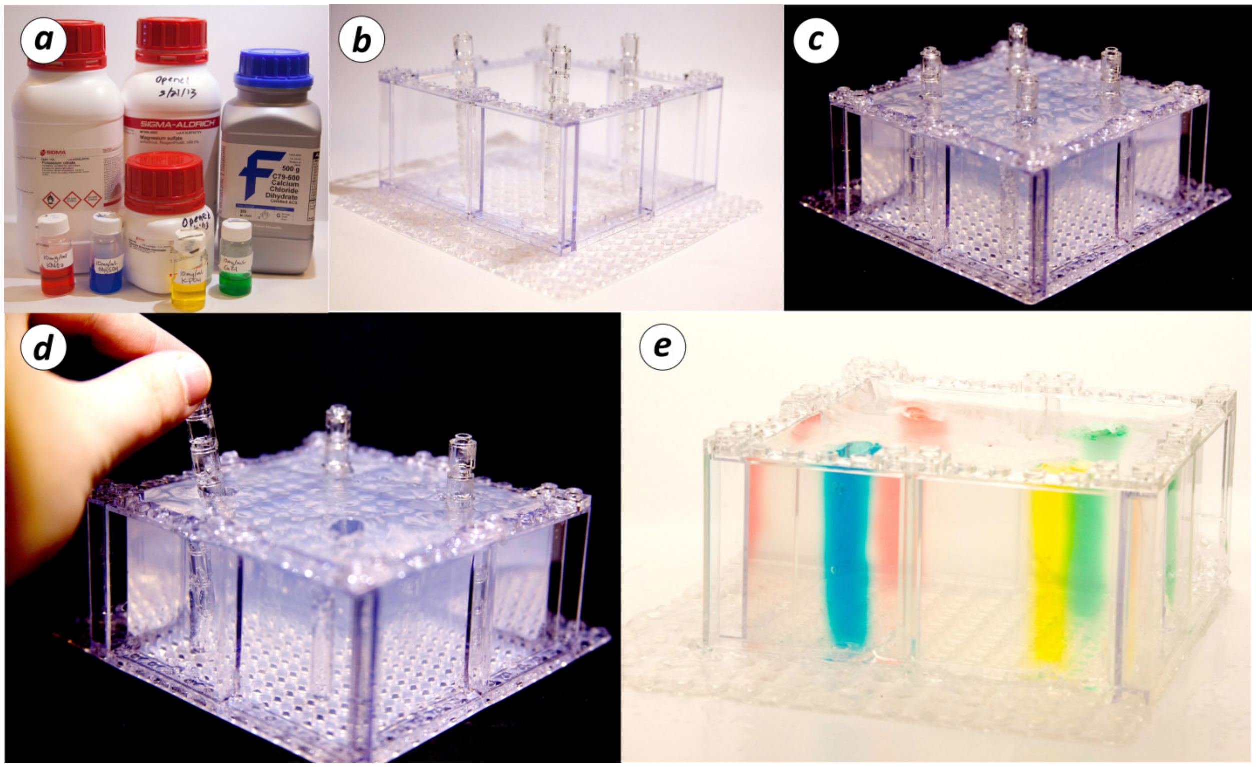

Figure S4. Snapshots of the procedure to produce 2-dimensional features (solid obstacles, air pockets and cylindrical chemical gradients) in a homogeneous gels by using LEGO bricks.

4. At this point, the LEGO box is cold from being the freezer for at least 2 hours. Spray the plastic box with 70% ethanol and reintroduce into the biosafety cabinet. Once the gel reaches near 35 °C slowly pour the gel in the box making sure the columns remain erect. It works best to add a small amount of

gel at a time. The gel required for this experiment is around 600 ml. Allow the experiment to remain in the biosafety cabinet overnight to allow the gel to fully set.

5. The next day, the columns are carefully removed. If the columns are stuck to the baseplate, a tweezers can be used to aid in the removal.
6. At this point the dyed solutions that were prepared in Table S1 can be added to each column or if doing a gas study they can remain empty. For the dye study, 2 ml of each dyed gel solution is pipetted into each column hole.
7. For either the dye experiment or gas experiment, the seed (Brassica Rapa in our case) can be planted into the center of the gel. Parafilm is used to seal the container before placing in the growth chamber.
8. Plant growth environments that combine solid features with air pockets and chemical gradients are produced according to the same procedure highlighted above.

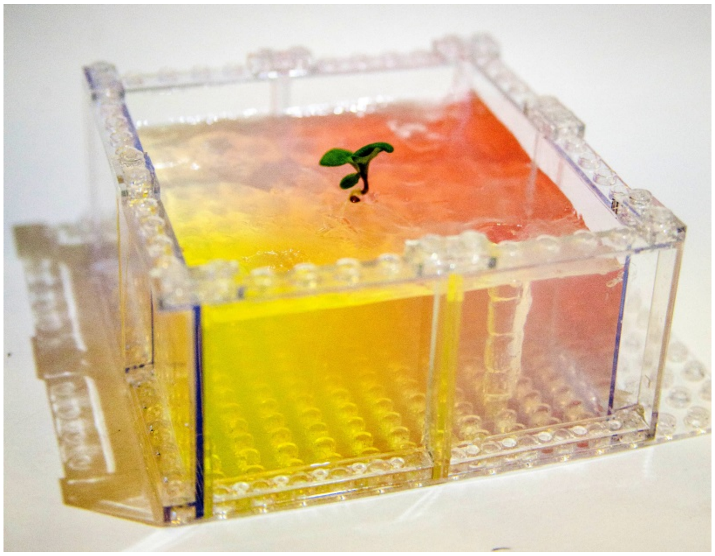

Figure S5. Photograph of a 3D plant growth environment based on LEGO bricks featuring three different types of heterogeneities: a solid barrier (top left), an air pocket (bottom right) and two different cylindrical chemical gradients (top right and bottom left)

<u>Steps 1-1: Modular plant experiment</u>

1. Modular plant experiments are based on the same procedures (i.e., templating) described above for the fabrication of heterogeneities in the gel.

<u>Steps 1-1: Scaled up plant experiments</u>

1. Scaled up plant experiments were produced with the following LEGO bricks, following the same procedures outlined above.
   Wall Element – TR 1X2X3 (LEGO Element ID: 6010737)
   Wall Element – TR 1X2X2 (LEGO Element ID: 4113028)
   Wall Element – TR 1X4X3 (LEGO Element ID: 4558208)
   Wall Element – TR 1X6X5 (LEGO Element ID: 4504229)
   Plate 1X2 (LEGO Element ID: 4167842)
   Plate 8X8 (LEGO Element ID: 4210802)
   X-large plate (LEGO Element ID: 4294466)
   Round brick (LEGO Element ID: 3006840)

CALCULATION OF SMALLEST POSSIBLE LEGO-BASED ENVIRONMENT

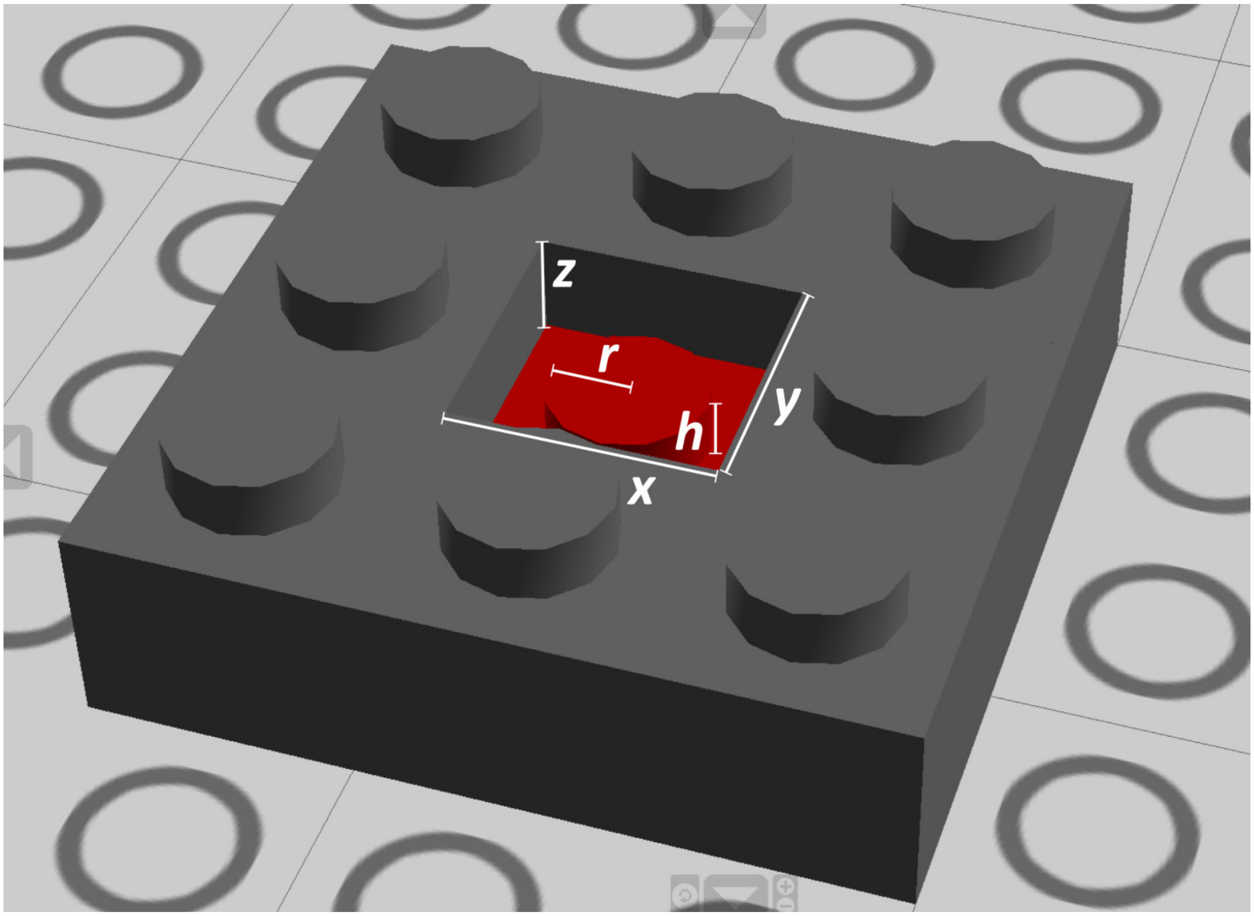

Figure S6. Depiction of the smallest LEGO-based environment.

Volume is $(x{\cdot}y{\cdot}z)-(\pi\cdot r{\cdot}h)$ = (0.8*0.8*0.6)-(3.14*0.25^2*0.17) = 0.35 cm^3.

LIMITATIONS AND OPEN QUESTIONS

While LEGO offers a remarkable set of assets for the design of environments for the growth of cm-scale organisms and systems of organisms, it does have limitations. Some of these limitations are intrinsic, while others can be plausibly overcome with more design work or by complementing this LEGO-based approach with other techniques. We here review the limitations of our approach.

1. The approach is not exactly boundless in that it is bound by the supply of pieces that LEGO provides, over which the scientist has no control over. The general dimensions and backward compatibility of LEGO bricks will be most likely preserved. It is not guaranteed, however, that all currently produced transparent bricks will be always in production. We do not believe this to be a major concern. The number of structures that can be produced with minimal subsets of the existing catalogue is staggering. Furthermore, any such concern is strongly alleviated by the progress in 3D printing.

Additive manufacturing could be easily used to compensate for the limitations in the choices of LEGO bricks.

2. The geometry of the LEGO system is based on right angles.
3. The delivery of individual pieces can take as long as three months, when ordering from the United States. As we mentioned before, other options (e.g., Ebay) exist that provide much faster delivery.
4. As we mentioned before, while a large number of LEGO bricks are autoclavable, there is a much larger range of brick types that are colored, composed of ABS plastic and thereby not autoclavable. Those bricks can be used for biological experiments, provided that they are sterilized by ethanol and/or bleach.
5. Producing a LEGO structure, such as a box, does not necessarily result in a liquid tight design. In the case of the gels, some leakage did occur during the experiments. Methods such as supergluing the brinks together could be done but result in a loss in future mobility of those bricks.

FAILED EXPERIMENTS

1. Acetone is not an effective method to reverse superglued LEGO bricks.
2. Wrapping cling wrap around the outside of the LEGO brick environment did not ultimately reduce gel leakage.
3. Dipping the entire LEGO brick environment mold in liquid gel did not close cracks and did not reduce gel leakage.
4. Gel concentrations below 3 g/l of Phytagel did not result in air-filled columns that remained intact for more than a day.
5. In a few experiments, the plant did not penetrate the gel and instead grew only on the surface of the gel. This can often be prevented if the seed is slightly submerged in the gel when planted or gel is used to encapsulate the seed.
6. In a few experiments, the root was capable of going between the LEGO brick cracks and thus finding its way outside the LEGO environment.
