# Supplementary figures and images for: LEGO® Bricks as Building Blocks for Centimeter-Scale Biological Environments: The Case of Plants
Source: PLoS One. 2014 Jun 25;9(6):e100867. doi: 10.1371/journal.pone.0100867 (PMC4071033; doi:10.1371/journal.pone.0100867)

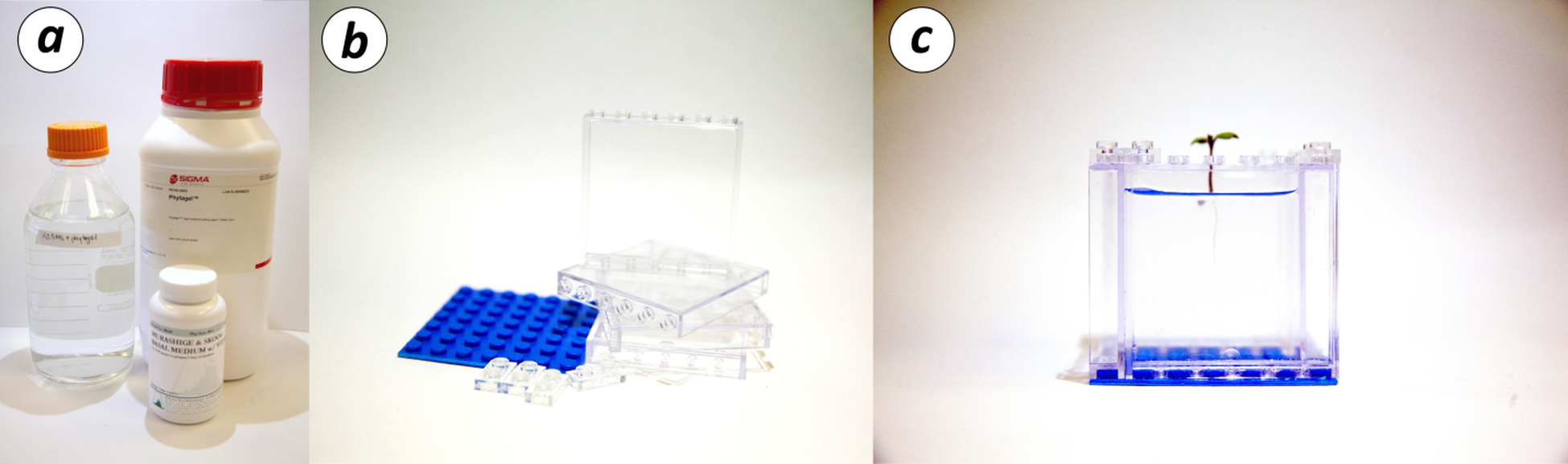

Supplement: Figure S1 — Summary snapshots of the assembly of a basic LEGO-based plant growth environment. (TIF) [file pone.0100867.s002.tif]

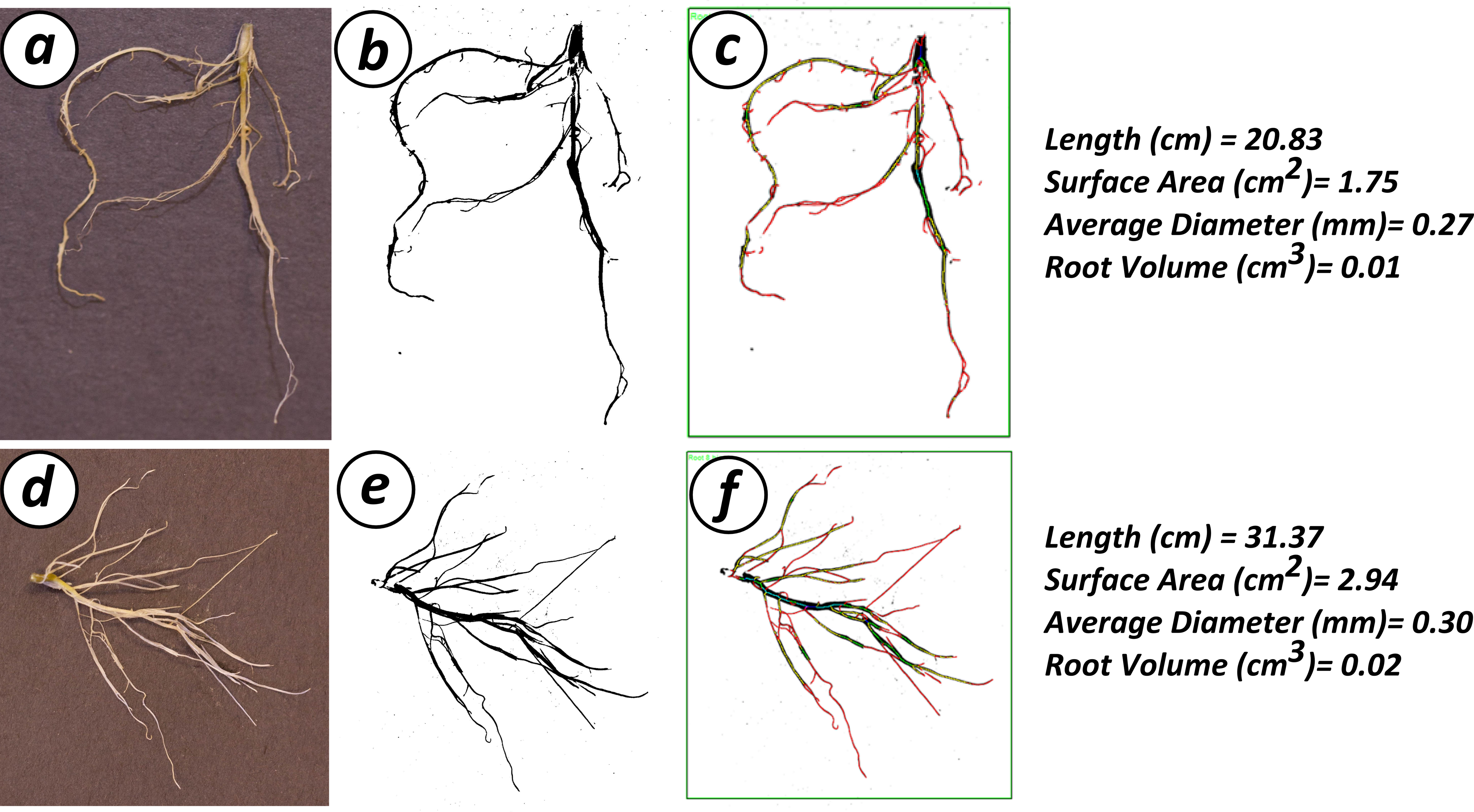

Supplement: Figure S2 — Summary snapshots of steps for root analysis using WinRhizo of two brassica rapa roots grown in LEGO-based plant growth environment. (TIF) [file pone.0100867.s003.tif]

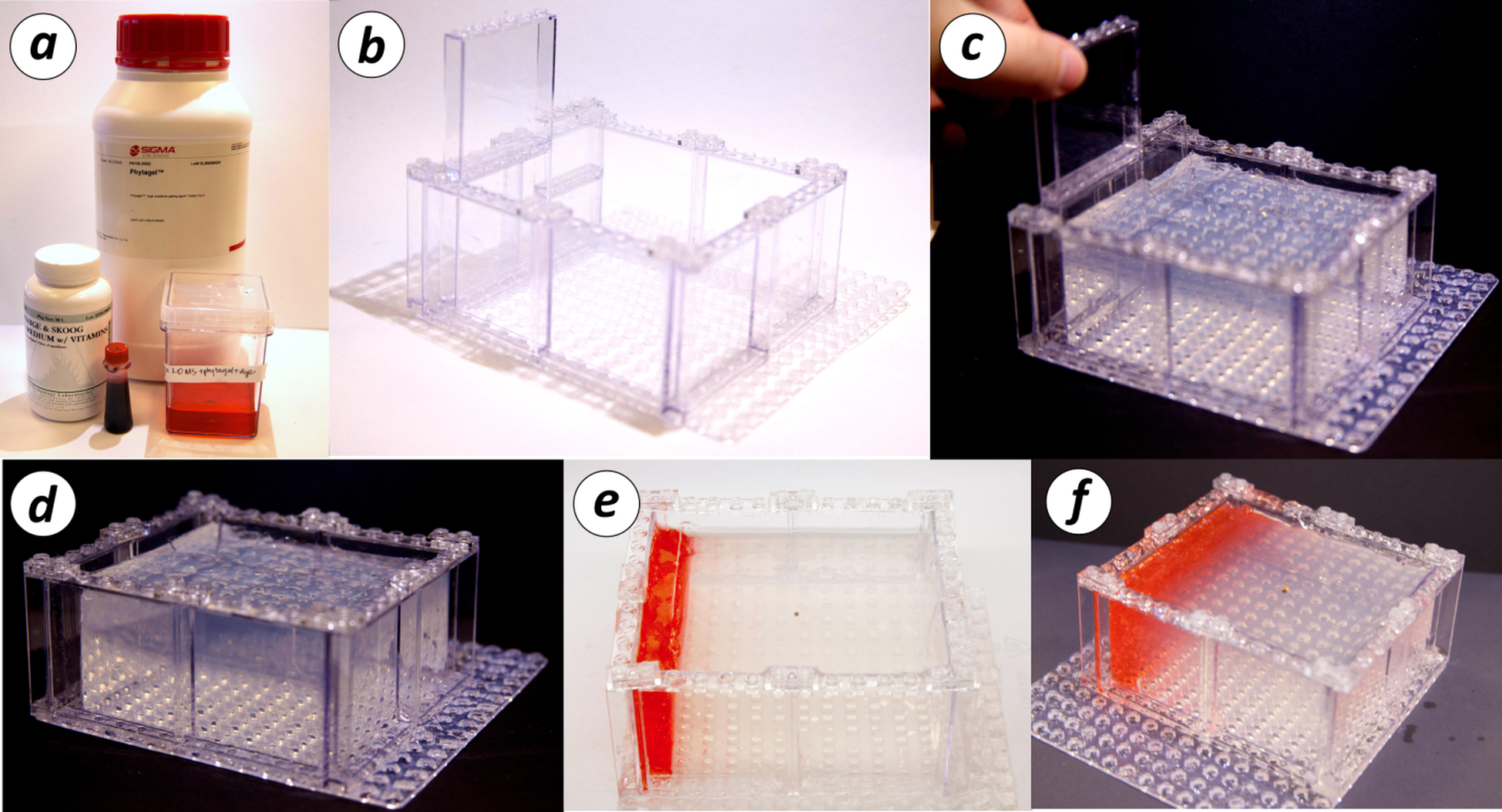

Supplement: Figure S3 — Snapshots of the procedure to produce linear features (solid obstacles, air pockets and chemical gradients) in a homogeneous gel by using LEGO bricks. (TIF) [file pone.0100867.s004.tif]

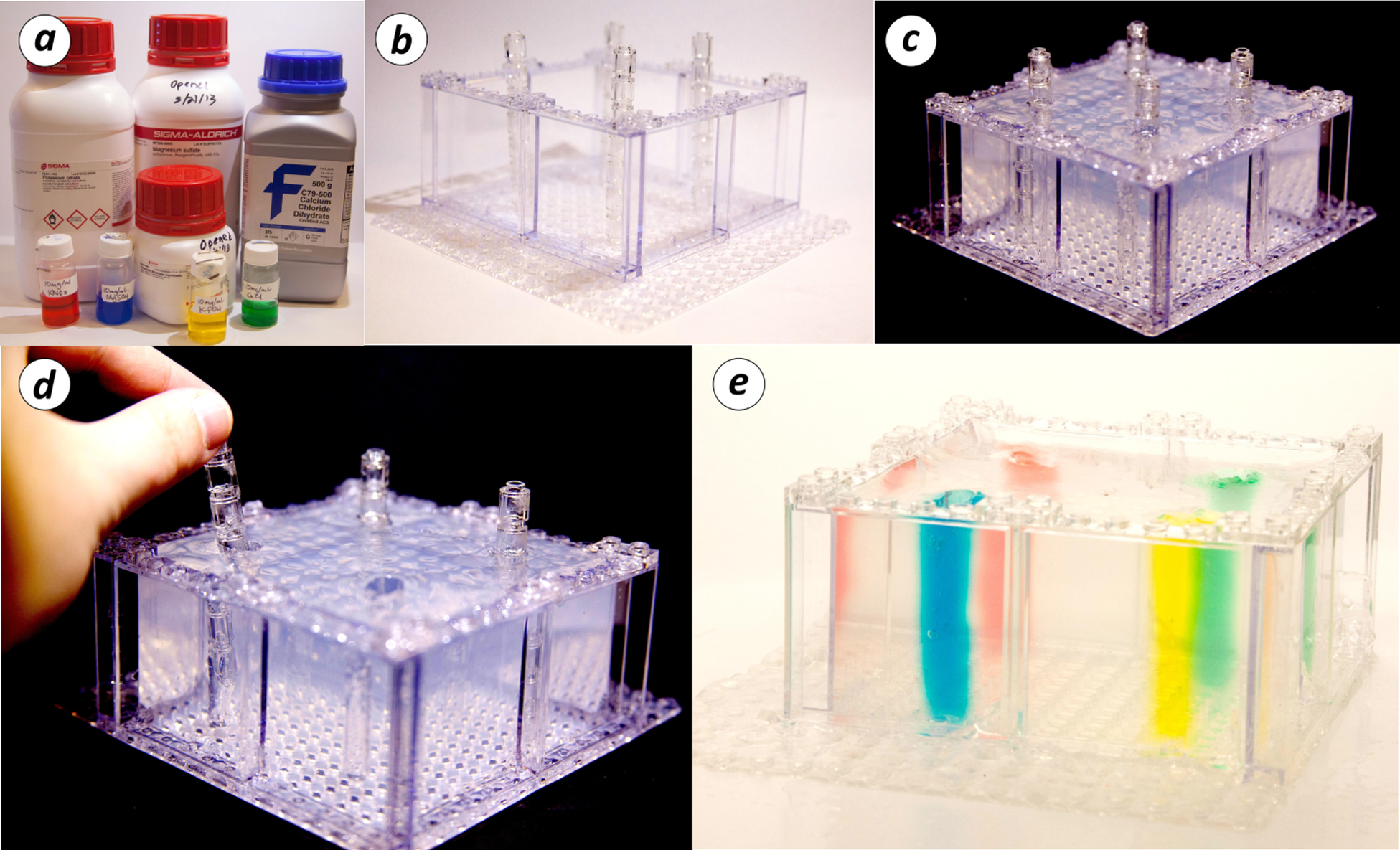

Supplement: Figure S4 — Snapshots of the procedure to produce 2-dimensional features (solid obstacles, air pockets and cylindrical chemical gradients) in a homogeneous gels by using LEGO bricks. (TIF) [file pone.0100867.s005.tif]

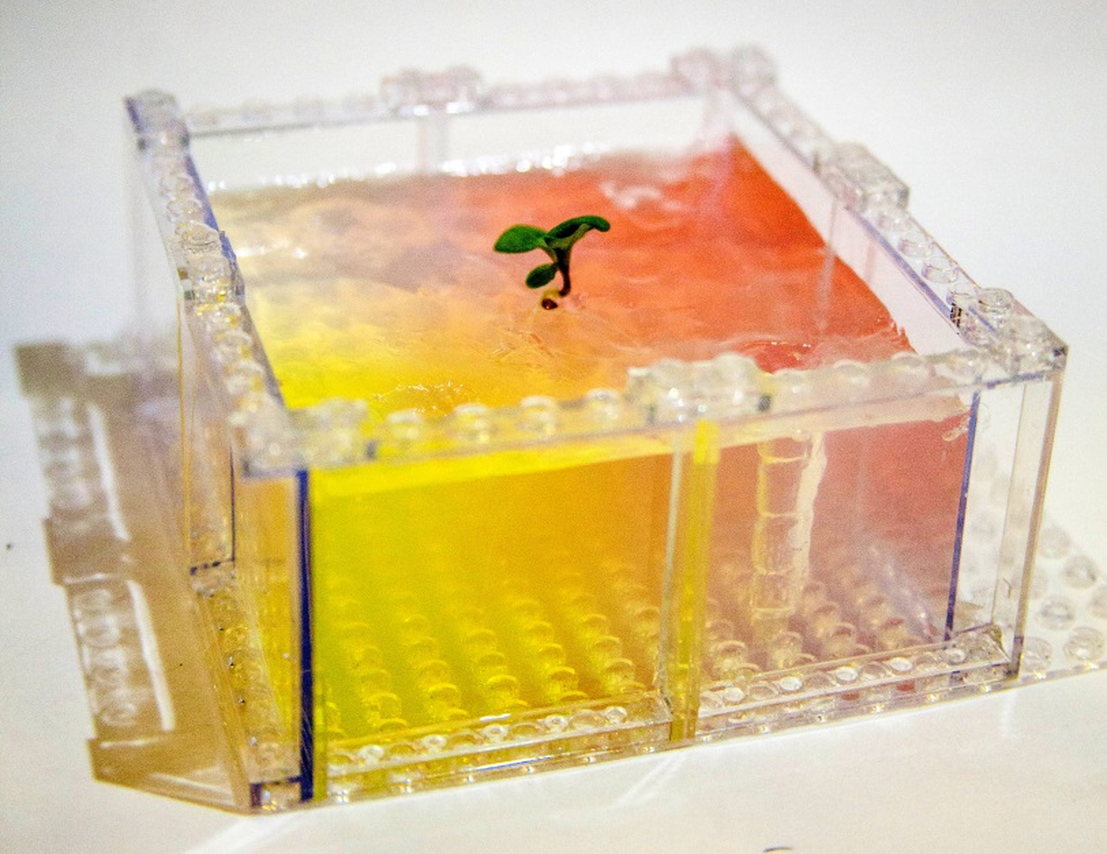

Supplement: Figure S5 — Photograph of a 3D plant growth environment based on LEGO bricks featuring three different types of heterogeneities: a solid barrier (top left), an air pocket (bottom right) and two different cylindrical chemical gradients (top right and bottom left). (TIF) [file pone.0100867.s006.tif]

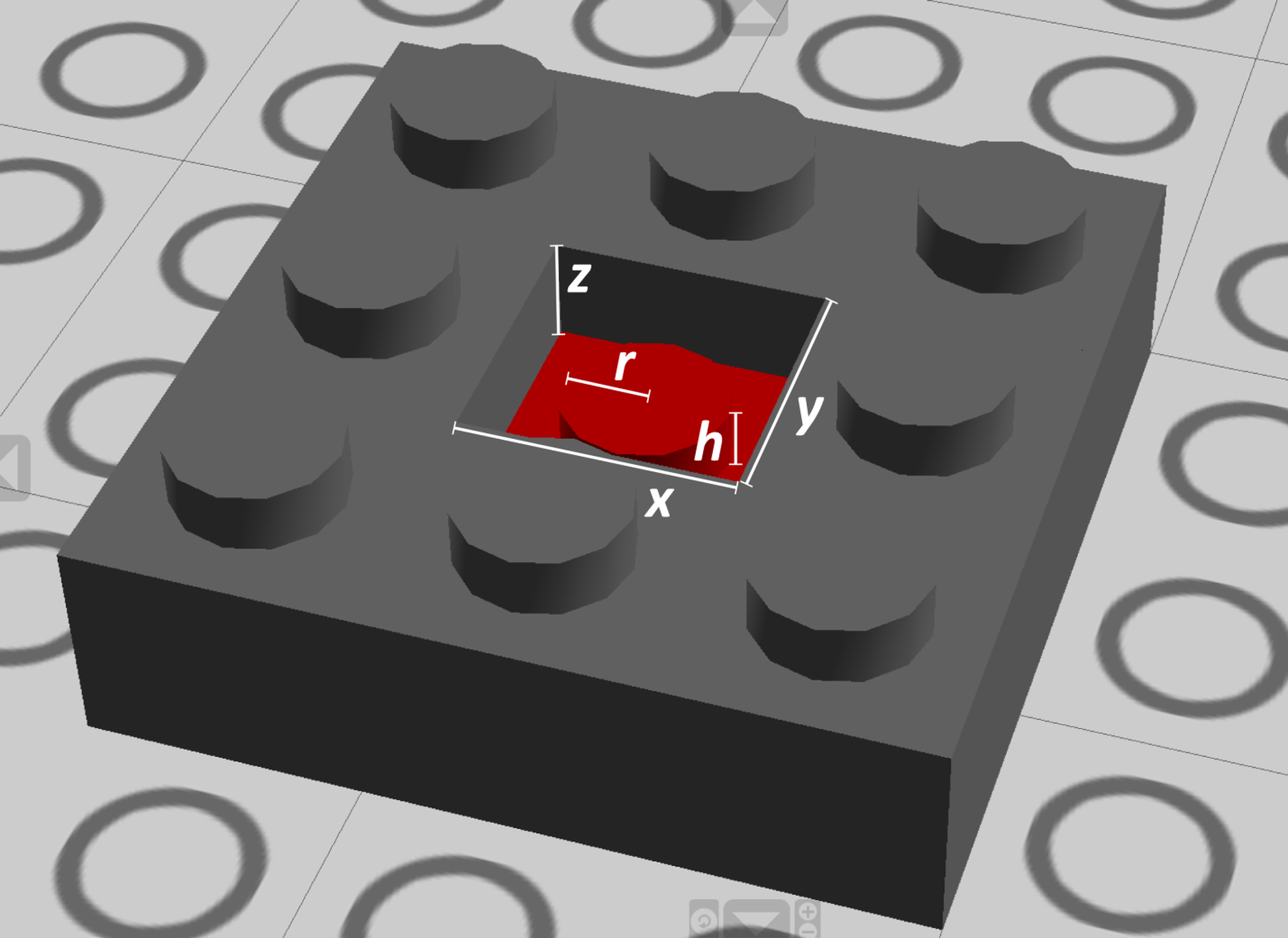

Supplement: Figure S6 — Depiction of the smallest LEGO-based environment. (TIF) [file pone.0100867.s007.tif]
