# Supplementary material for: LEGO® Bricks as Building Blocks for Centimeter-Scale Biological Environments: The Case of Plants
Source: PLoS One. 2014 Jun 25;9(6):e100867. doi: 10.1371/journal.pone.0100867 (PMC4071033; doi:10.1371/journal.pone.0100867)
Supplement: Table S1 — Preparation of salt solutions for cylindrical dye experiment. (PDF) [file pone.0100867.s010.pdf]

LEGO® Bricks As Building Blocks For Centimeter-Scale Biological Environments: The Case Of Plants

KARA R. LIND[†], TOM SIZMUR[†‡§], SAIDA BENOMAR[†‡], ANTHONY MILLER[†‖], LUDOVICO CADEMARTIRI[†‡⊥*]

† Department of Materials Science & Engineering, Iowa State University, Ames, IA, 50011
‡ Ames Laboratory, US Department of Energy, Iowa State University, Ames, IA, 50011
‖ Department of Agronomy, Iowa State University, Ames, IA, 50011
⊥ Department of Chemical & Biological Engineering, Iowa State University, Ames, IA, 50011
§ current address: Department of Sustainable Soils and Grassland Systems, Rothamsted Research, Harpenden, Heartfordshire, AL5 2JQ, United Kingdom

Author to whom correspondence should be addressed: lcademar@iastate.edu

TABLE S1

Solution #	Salt	Additional components	Dye
**1**	100 mg potassium phosphate	10 ml milliQ water<br>30 mg phytagel	Green food coloring<br>30 µl
**2**	100 mg potassium nitrate	10 ml milliQ water<br>30 mg phytagel	Yellow food coloring<br>30 µl
**3**	100 mg calcium chloride	10 ml milliQ water<br>30 mg phytagel	Red food coloring<br>30 µl
**4**	100 mg magnesium sulfate	10 ml milliQ water<br>30 mg phytagel	Blue food coloring<br>20 µl

Table S1: Preparation of salt solutions for cylindrical dye experiment.
